# Retraction notice to “Deregulated expression of Elastin Microfibril Interfacer 2 (EMILIN2) in gastric cancer affects tumor growth and angiogenesis.” [Matrix Biol. Plus 6–7 (2020) 100029]

**DOI:** 10.1016/j.mbplus.2025.100184

**Published:** 2025-11-12

**Authors:** Eva Andreuzzi, Albina Fejza, Alessandra Capuano, Evelina Poletto, Eliana Pivetta, Roberto Doliana, Rosanna Pellicani, Andrea Favero, Stefania Maiero, Mara Fornasarig, Renato Cannizzaro, Renato V. Iozzo, Paola Spessotto, Maurizio Mongiat

**Affiliations:** a Department of Research and Diagnosis, Division of Molecular Oncology, Centro di Riferimento Oncologico di Aviano (CRO) IRCCS, Italy; b Department of Clinical Oncology, Experimental Gastrointestinal, Centro di Riferimento Oncologico di Aviano (CRO) IRCCS, Italy; c Department of Pathology, Anatomy, and Cell Biology and the Cancer Cell Biology and Signaling Program, Sidney Kimmel Medical College at Thomas Jefferson University, Philadelphia, PA, USA

This article has been retracted: please see the Elsevier policy on article withdrawal (https://www.elsevier.com/about/policies-and-standards/article-withdrawal).

This article has been retracted at the request of the Editor-in-Chief.

After publication, an anonymous reader raised concerns to the journal about the similarity of the merge image panels for Patients 2 and 3 in Figure 2A (now on PubPeer: https://pubpeer.com/publications/9FF57B7D611B428924DB2816CF6C02). The Editorial team investigated the images in question, and subsequent inspection with image analysis software identified multiple duplicated image panels.

The images in panels EMILIN2, CD31, and Merge in Figure 2A for Patients 2 and 3 have an area of overlap where the field of view was shifted. There is also concern about the difference in intensity between the overlapping areas of Patients 2 and 3, particularly in the EMILIN2 panels.

The corresponding author acknowledged the errors and provided raw data and corrected figures. He indicated that Figure 2A inadvertently included data from Patient 11 instead of Patient 26. The updated figure relabeled ‘Patient 1’, ‘Patient 2’, and ‘Patient 3’ as Patient 12, Patient 11, and Patient 26, respectively. He also noted that the replacement images retained the same contrast adjustments as originally presented and that contrast adjustments to reduce background noise and to improve clarity were applied uniformly and did not alter the underlying data or scientific interpretation of the results.

The panel for Gastric cancer and EMILIN2 in Figure 1A is duplicated in the EMILIN2 panel for Patient 3 in Figure 3A.

The corresponding author stated that Figure 3A mistakenly displayed the EMILIN2 immunofluorescence for Patient 15 that appears in the Gastric cancer panel in Figure 1A in place of that for Patient 17. An updated version of Figure 3A was provided with a new EMILIN2 panel for Patient 17, and ‘Patient 1’, ‘Patient 2’, and ‘Patient 3’ were relabeled as Patient 18, Patient 12, and Patient 17, respectively. However, while the new image panels were labeled Patient 17 (previously ‘Patient 3’), the updated legend referred to Patient 15 rather than 17.

The Editorial team was concerned by the corresponding author’s response indicating images for Patients 11, 15, 17, and 26, rather than for Patients 2 and 3. Using ‘Patients 1, 2, and 3’ to label panels in Figures 2 and 3 when they were not the same patients is misleading. This could lead readers to assume that the samples from Patients 1, 2, and 3 in both figures were from the same three patients rather than from at least five distinct patients; note that Patient 12 was shown as Patient 1 in the published Figure 2A and as Patient 2 in Figure 3A.

The description of the number of patients enrolled in this study and the number evaluated in the cohort was inaccurate. The Materials and Methods stated that 19 patients were consecutively enrolled, but Table 1 suggested that the cohort was 25. The corresponding author explained that the patient cohort was expanded from 19 during the study and that the study consecutively enrolled 29 patients. Four were excluded due to limited tissue amount or poor quality, leaving 25 patients.

Although the study included 25 patients, the inclusion of 27 data points rather than 25 in the Pearson correlation analyses in Figures 2B and 3B raised further concerns. Both legends indicate that the dots correspond to the patients.

Given the issues noted above, the Editorial team no longer has confidence in the validity or integrity of the article. The scientific community takes a very strong view on this matter, and apologies are offered to readers of the journal that this was not detected during the submission process.

Corresponding author Maurizio Mongiat disagrees with the retraction and disputes the grounds for it. The co-authors did not respond to the retraction notice.

